# Mitochondrial Dysfunction in Parkinson’s Disease—Cause or Consequence?

**DOI:** 10.3390/biology8020038

**Published:** 2019-05-11

**Authors:** Chun Chen, Doug M. Turnbull, Amy K. Reeve

**Affiliations:** 1 Wellcome Centre for Mitochondrial Research, Institute for Neuroscience, Newcastle University Institute for Ageing, Newcastle University, Newcastle upon Tyne, NE2 4HH, UK; c.chen20@ncl.ac.uk (C.C.); doug.turnbull@newcastle.ac.uk (D.M.T.); 2 Newcastle University LLHW Centre for Ageing and Vitality, Newcastle University, Newcastle upon Tyne, NE2 4HH, UK

**Keywords:** Parkinson’s disease, mitochondria, ageing, neurodegenerative disease

## Abstract

James Parkinson first described the motor symptoms of the disease that took his name over 200 years ago. While our knowledge of many of the changes that occur in this condition has increased, it is still unknown what causes this neurodegeneration and why it only affects some individuals with advancing age. Here we review current literature to discuss whether the mitochondrial dysfunction we have detected in Parkinson’s disease is a pathogenic cause of neuronal loss or whether it is itself a consequence of dysfunction in other pathways. We examine research data from cases of idiopathic Parkinson’s with that from model systems and individuals with familial forms of the disease. Furthermore, we include data from healthy aged individuals to highlight that many of the changes described are also present with advancing age, though not normally in the presence of severe neurodegeneration. While a definitive answer to this question may still be just out of reach, it is clear that mitochondrial dysfunction sits prominently at the centre of the disease pathway that leads to catastrophic neuronal loss in those affected by this disease.

## 1. Introduction

Mitochondrial dysfunction was proposed to be an integral player in the development of Parkinson’s disease (PD) nearly 40 years ago, and since those initial discoveries, evidence of the role it may play in this neurodegeneration continues to increase. There is now evidence to suggest a role not only for a loss of mitochondrial function in terms of ATP provision and calcium buffering capacity, but also the degradation of these organelles through mitophagy and the interaction of mitochondria with other organelles and proteins in this disease. Furthermore, over the last 30 years, our understanding of the molecular pathways involved in the development of PD has grown immensely. These developments have been driven in part by improved genetic techniques allowing large-scale screens to be performed, but also by the speed with which disease-causing genes can be identified within affected families. In light of this, the number of genes associated with both early onset familial PD and the sporadic form of the disease continues to increase. This growing body of work continues to highlight pathways likely to be important for the development of PD, many of which centre around the function of mitochondria.

## 2. Mitochondrial Function

### 2.1. Mitochondrial Respiratory Chain

The first link between mitochondrial dysfunction and PD became evident in 1983, when 1-methyl-4-phenyl-1,2,3,6-tetrahydropyridine (MPTP) was found to cause parkinsonian-like symptoms in intravenous drug users [1,2]. Once MPTP penetrates the blood-brain barrier, this lipophilic compound is bio-transformed into its toxic form 1-methyl-4-phenylpyridinium (MPP+) by glial monoamine oxidase (MAO) [3]. MPP+ specifically interferes with the activity of respiratory chain (RC) complex I (NADH: Ubiquinone oxidoreductase) in dopaminergic (DA) neurons, causing selective neurodegeneration in both human and mouse substantia nigra (SN) [2,4,5]. Since this original study, several environmental toxins which also are capable of potent inhibition of mitochondrial complex I have been implicated in the epidemiology of sporadic PD. This raised awareness of the association of environmental exposure with the risk of developing PD later in life, via damage of RC function and increased oxidative stress (reviewed in [6,7]). These neurotoxins include rotenone, a wide spectrum insecticide [8]; organochlorine pesticides, such as chloripyrifos and permethrin, and macromolecular solvents such as 1-trichloromethyl-1,2,3,4-tetrahydro-β-carboline (TaClo) and trichloroethylene (TCE) (reviewed in [6]). However, the toxicity of these molecules may not simply lie in their inhibition of Complex I, but may also be related to exposure level, and their effect on other cellular processes, for example intracellular dopamine oxidation [9,10,11,12].

**Figure 1:**
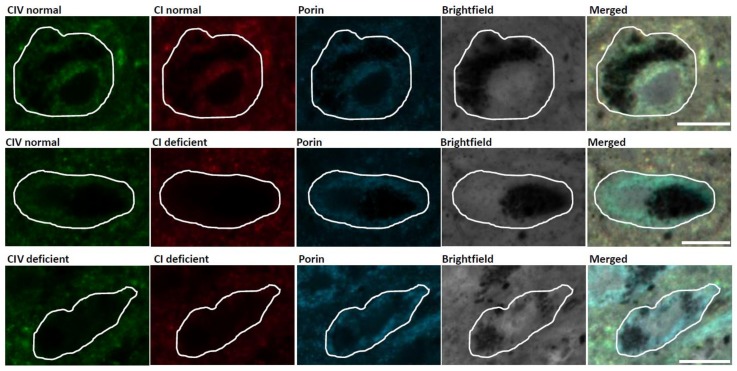
Mitochondrial respiratory chain (RC) deficiency in substantia nigra (SN) neurons of an individual with Parkinson’s disease (PD). Immunofluorescent images demonstrating SN neurons with normal Complex I (CI) and Complex IV (CIV) expression, Complex I deficiency and normal Complex IV expression, and deficiency of both Complex I and Complex IV. Scale bar, 20 µm.

In post-mortem studies, varying degrees of Complex I (Figure 1) and complex II (succinate dehydrogenase, SDH) deficiency have been found in individual SN neurons from PD patients (~60% Complex I and ~65% Complex II deficiency [13]). A widespread decrease in complex I expression has also been observed in multiple brain regions in PD, including hippocampus, putamen, and pedunculopontine nucleus [14,15]. While immunofluorescent (protein expression) [13] and COX (Complex IV, cytochrome *c* oxidase)/SDH (enzyme activity) [16,17] assays have identified that 25–30% of SN neurons show a deficiency for Complex IV (Figure 1). This deficiency commonly presents alongside the aforementioned Complex I or Complex II deficiency in both PD and normal ageing [13,18]. It is possible that the predisposition for Complex I deficiency in SN neurons is due to a low reserve for Complex I function [19]. Furthermore, the high number of mitochondrial DNA (mtDNA) encoded subunits required for Complex I assembly confers that this complex is more likely to be affected by pathogenic mtDNA mutations in the susceptible neuronal populations. However, the causal role of Complex I deficiency in SN neuronal loss remains unclear. The loss of Complex I seems to be better tolerated than that of other RC subunits, given that defects in the expression of this complex are commonly detected in surviving SN neurons in PD cases and aged matched individuals, as well as in neurons from multiple other regions [13,14,18]. Mutations in the gene encoding the mitochondrial polymerase, *POLG*, in mitochondrial disease patients cause accumulated mitochondrial dysfunction similar to that detected in PD and ageing [20]. Complex I-negative neurons are found in infant *POLG* patients, before the prominent loss of neurons, which could further support a less detrimental effect of Complex I deficiency on neuronal survival, compared to the loss of other RC complexes [21].

Single neuron studies have attempted to interrogate the relationship between mitochondrial dysfunction and the formation of alpha-synuclein pathology [14,22]. Significantly higher expression of Complex I and Complex IV was found in SN neurons with alpha-synuclein pathology [22], whilst the presence of alpha-synuclein aggregation was less frequent in Complex I deficient SN neurons [14,22]. Both post-mortem studies suggested that alpha-synuclein pathology and abnormal RC function may be two independent factors in PD pathogenesis.

### 2.2. Mitochondrial DNA Defects

The potential consequences of clonally expanded mtDNA deletions and related biochemical defects on the survival of SN neurons have been investigated in several studies. Indeed, somatic mtDNA deletions reaching 50% have been described at the single SN neuron level in both PD and healthy ageing, with much higher levels in COX-deficient cells [16,17]. These deletions were found to be of various sizes (~2000 to ~9500bp), located within the major arc of mtDNA, and involve both tRNA and mitochondrial RC protein genes [20]. It is therefore evident that these multiple mtDNA deletions are somatically acquired, with the clonal expansion of different-sized deletions within each individual SN neuron, thus yielding a wide spectrum of mtDNA deletion breakpoints within an individual [20]. A recent post-mortem study revealed an accumulation of mtDNA deletions with advancing age in individual SN neurons, whilst such a trend did not occur in cortical or cerebellar neurons and the deletion load was much lower in these neuronal populations [23].

Although the mechanism for the formation of mtDNA deletions is still under debate, the necessity of mtDNA replication activity in deletion formation [24] was challenged by the absence of 3′-repeat retention in some of the detected mtDNA deletion species in SN neurons [20]. According to the “slipped-replication” theory, a single stranded loop occurs between 3′5′ direct repeats which is then exposed and degraded during mtDNA replication, leading to the occurrence of deletions that are often located within the major arc of mtDNA [24]. Krishnan et al. proposed another possibility that mtDNA deletion formation may initiate from 3′–5′ exonuclease activity during the repair of damage to mtDNA in the SN neuronal population [25]. MtDNA damage leads to the formation of double strand breaks (DSB), and misrepair of such breaks could cause the loss of several kilobases of mtDNA on both strands. Single strand mtDNA generated from this process would then anneal with homologous sequences, resulting in the double-stranded deleted molecule with both a 5′ and 3′ repeat [25]. The relationship between DSB repair and the generation of mtDNA deletions has been supported by several studies using inducible mitochondrial-targeted restriction endonuclease, *Pst*I, to trigger mtDNA DSB. Multiple mtDNA deletions were found in cultured *Pst*I neurons [26] and in DA neurons of a *Pst*I mouse model [27]. These mice also developed nigrostriatal degeneration and PD-related behavioral phenotypes [27]. Later, Moraes’ team reported an acceleration effect of mtDNA DSB on neuronal ageing through reactive oxygen species (ROS) induced damage [28]. The nature of deletion formation through repair could therefore be regarded as an inevitable cost of mtDNA self-rescue/regulation against oxidative damage. This fits well with the strikingly high deletion load of mtDNA detected in aged SN neurons of both healthy individuals and PD patients [20,23], as ROS-induced mtDNA damage advances with ageing.

In addition to the higher mtDNA deletion load in the SN than other aged brain regions, such as the basal ganglia, cortical areas and cerebellum [23,29,30], Dolle et al. also revealed a age-related increase of mtDNA copy number within individual SN neurons [23]. More importantly, the upregulation of mtDNA copy number also correlates with the increase in mtDNA deletion load within the SN neuronal population. The corresponding increase of mtDNA copy number with mtDNA deletion level, together with an enhancement of mitochondrial function, were mirrored in a study of DA neurons from a premature ageing model (POLG^D257A^ mice) [31]. These findings suggested an ability of age SN neurons to adapt to mitochondrial dysfunction via the maintenance of their mtDNA population. Failure of such regulatory mechanisms of mtDNA copy number is described in PD patients in several studies. Grunewald et al. suggested depletion of mtDNA copy number and low expression of an essential mtDNA nucleoid protein, mitochondrial transcription factor A (TFAM) in the SN neurons of PD patients [13]. However, another study showed a decrease in mtDNA wildtype copy number but not in total copy number, alongside a decreased correlation between mtDNA copy number and deletion level in PD neurons compared to healthy aged neurons [23]. Reduction of mtDNA copy number in the peripheral blood [32] and cerebrospinal fluid [33,34] has also been detected in patients with PD and neurodegenerative disease.

These studies all suggest that the formation and accumulation of high levels of mtDNA deletions leads to mitochondrial dysfunction that will have an impact on the health and survival of SN neurons. However, many of the reported findings are also found in normal ageing in the SN. Therefore, mtDNA alterations are certainly one cause of the mitochondrial deficiency detected within these neurons and will undoubtedly increase the vulnerability of these neurons to loss in PD, but the question still remains whether these defects are causative of PD itself.

### 2.3. ROS Production and Oxidative Stress

The accumulation of somatic mtDNA deletions and the subsequent development of RC deficiency within aged SN neurons may lead to impaired mitochondrial membrane potential (∆Ѱm), reduced synthesis of ATP, and an increase in release of ROS leading to oxidative stress [35]. A higher rate of electron flux due to an increase in respiratory capacity to adapt to high metabolic demands in SN neurons has also been thought to cause increased ROS generation. The increase in numbers of electrons being transferred would increase the probability of electron capture by O_2_ to generate superoxide before they reach COX [36]. In addition to the production and release of ROS from mitochondria, the process of dopamine metabolism by monoamine oxidase could also account for the accumulation of toxic oxidative species such as hydroxyl radicals [37]. Oxidative damage to mtDNA results in further deficiency in RC expression and creates a vicious circle of oxidative stress and bioenergetics failure. This “mitochondrial theory of ageing” has long been regarded as a rationale for mitochondrial changes with advancing age [38], which could contribute to the increase in SN neuronal vulnerability. However, this theory was challenged by more recent studies which manipulated the antioxidant capacity of several mouse models, finding impaired integrity of the mitochondrial genome which was not necessarily associated with an age-related phenotype or shortened lifespan (reviewed in [39]). Evidence from a premature ageing mouse model (POLG^D257A^ mutant) found that the generation of mitochondrial ROS or mtDNA damage did not increase with a high heteroplasmic level of mtDNA point mutation, and that there seems to be a threshold for mtDNA deletions to cause accelerated ageing [40].

In addition to mitochondrial ROS, other factors that may contribute to increased oxidative damage include iron accumulation and an increase in lipid peroxidation burden [41,42], while accumulation of hydroxyl and superoxide radicals due to a decline in glutathione content [43] have also been reported in the brain of PD patients. Recent studies also highlighted a direct toxicity of alpha-synuclein oligomers on mitochondria via induction of mitochondrial lipid peroxidation and oxidation of ATP synthase [44,45]. It was shown that alpha-synuclein leads to increased permeability of mitochondrial membranes and ROS production, ultimately leading to neuronal death [45].

### 2.4. Calcium Handling

Emerging evidence has suggested the importance of cytosolic calcium ions (Ca^2+^) in the regionally selective nature of neuronal loss in PD pathogenesis. Dopaminergic neurons in the SN exhibit autonomous pacemaking activity in order to maintain regular release of dopamine to the striatum, facilitated by CaV1.3 L-type calcium channels. Action potentials generated from this process are broad, with a relatively slow rate (2–10 Hz), promoting a slow rhythmic activity accompanied with a slow oscillation in cytosolic Ca^2+^. A relationship between Ca^2+^ oscillations and PD neuronal loss was initially proposed by experiments showing a decline in the number of calbindin (a Ca^2+^ binding protein), positive neurons in PD patients [46,47]. These slow Ca^2+^ oscillations promote the import of Ca^2+^ into the mitochondrial intermembrane space and matrix. Intramitochondrial Ca^2+^ is required for the activation of pyruvate dehydrogenase, isocitrate dehydrogenase and α-ketoglutarate (important enzymes in the tricarboxylic acid (TCA) cycle) and promotes RC function and ATP production. Mitochondrial sequestration of Ca^2+^ benefits synaptic neurotransmission in terms of assisting in the stabilization of the cytosolic Ca^2+^ concentration during vesicle exocytosis [48] and promoting the recovery of synaptic excitation after exocytosis [48,49]. The unique feature of CaV1.3 channels is that they open at relatively hyperpolarized ∆Ѱm. Even so, the lack of powerful intracellular buffering of Ca^2+^ from the endoplasmic reticulum (ER) in DA neurons drives a sustained Ca^2+^ flux to enter mitochondria. This is able to promote ATP synthesis in the absence of a strong energy demand [50,51]. Ca^2+^ overload may force the opening of mitochondrial permeability transition pore (mPTP), leading to retrograde electron flux through the electron transport chain, resulting in increased ROS production, release of cytochrome *c*, and activation of apoptosis [52,53]. The increase in mitochondrial oxidative stress has also been identified in other neuronal populations, including the dorsal motor nucleus of the vagus (DMV) and the locus coeruleus (LC), which also express CaV1.3 channels. DA neurons in the ventral tegmental area (VTA) which have modest CaV1.3 channel currents however, demonstrate low mitochondrial oxidative stress and an improved ability to buffer cytosolic Ca^2+^ with ageing [54,55]. All of these indicate that CaV1.3 channels cause extra oxidative burden on mitochondria that may contribute to the selectively vulnerability of SN neurons (reviewed in Reference [56]). A recent report showed depletion of alpha-synuclein prevents the elevation of cytosolic Ca^2+^ concentration induced by MPP+ in mouse SN neurons, suggesting synergistic detrimental effects of these two pathological aspects in PD neurodegeneration [57].

### 2.5. Two Novel Familial PD Genes Associated with Mitochondrial Function

#### 2.5.1. VPS35

A novel autosomal dominant PD related pathogenic mutation was identified in the vascular protein sorting associated protein 35 (*VPS35*, PARK17, OMIM 614203) gene [58,59], and has been linked to impaired RC complex I and II subunit assembly and activity [60]. The VPS35 protein is a core subunit of the cargo recognition subcomplex that mediates the sorting, trafficking, and endocytosis of synaptic vesicles [35]. The *VPS35* mutation-induced mitochondrial dysfunction has been suggested to be a consequence of impaired mitochondrial fusion, tipping the balance of the fission and fusion cycle toward excessive fission. VPS35 promotes the degradation of mitochondrial E3 ubiquitin ligase1 (MUL1), and stabilizes mitofusin 2 (MFN2) preventing its degradation by MUL1 [61]. VPS35 deficiency impairs the regulatory machinery of mitochondrial dynamics via degradation of MFN2 by MUL1 in DA neurons, leading to mitochondrial dysfunction and fragmentation, which may underlie related PD pathogenesis [61]. Furthermore, a recent report has shown prominent changes in dopaminergic synaptic function, including increased dopamine turnover, loss of the dopamine transporter (DAT) and increased vesicular monoamine transporter 2 (VMAT2) expression in a *VPS35* knock-in mouse model [62].

#### 2.5.2. CHCHD2

Mutations in the coiled-coil-helix-coiled-coil-helix domain containing 2 (*CHCHD2*, PARK22, OMIM 616244) gene have recently been associated with autosomal dominant PD in Japanese families [63]. The role of *CHCHD2* as a PD-related gene was confirmed by genetic screening of affected Chinese families [64], however, studies in Caucasians, south Italians [65,66], and Brazilians [67] did not support its causative role in PD. It has been proposed that the localization of the CHCHD2 protein in the mitochondrial intermembrane space is beneficial to the maintenance of mitochondrial cristae structure and integrity of the mitochondrial RC [68,69]. Inhibition of CHCHD2 was found to impair mitochondrial Complex III (cytochrome *bc_1_* complex) and Complex IV activity, leading to decreases in OXPHOS activity and increased oxidative damage [69,70]. These mitochondrial structural and biochemical defects have also been shown in fibroblasts generated from a PD patient who carried a homozygous variant in *CHCHD2* [71]. In addition to the mitochondrial structural and biochemical defects, *Drosophila* carrying the *CHCHD2* mutations identified in patients also manifest motor symptoms and DA neurodegeneration [69].

### 2.6. Does Mitochondrial Dysfunction Drive the Development of Neurodegeneration in PD?

It is evident from other conditions, for example, mitochondrial disorders associated with mutations in the polymerase gamma gene, *POLG*, that a primary mitochondrial defect is sufficient to cause loss of SN neurons (and other neuronal populations) [18,72]. Furthermore, such mutations are often associated with the development of PD-like symptoms (reviewed in Reference [73]). However, attempting to disentangle “cause and effect” in the contribution of mitochondrial dysfunction to the pathogenesis of PD is difficult, since many of the changes we detect in those with PD are also present in healthy aged individuals. The mitochondrial defects present in these individuals often exist in the absence of cell loss or parkinsonian symptoms. For example, equivalent levels of mtDNA deletions are detected in SN neurons from individuals with PD and healthy controls [16,17] and neurons showing deficiencies for both Complex I and Complex IV are also detected in both instances [18]. Many of the processes described above initiate changes in other pathways and thus activate well defined responses which exist to mitigate to these changes, for example, ROS and antioxidants. Therefore, it might be suggested that in PD it is the ability of neurons to respond to mitochondrial functional changes that is impaired. Thus, neurons become more sensitive to changes in other pathways, which ultimately leads to their degeneration and loss.

## 3. Mitochondrial Turnover

### 3.1. Mitophagy

Mitophagy contributes to mitochondrial quality control by the selective clearance of damaged mitochondria, targeting an entire mitochondrion or fission fragmented mitochondria for autophagy. As a specialized form of autophagy, mitophagy comprises three necessary stages: the recognition of impaired mitochondria, the formation of autophagic membranes around the target, and the fusion of the mitoautophagosome with a lysosome (reviewed in Reference [74] and Reference [75]). Two familial PD-associated proteins, phosphatase and tensin homolog (PTEN)-induced putative kinase 1 (PINK1, PARK6, OMIM 608309) and E3 ubiquitin ligase, Parkin (PARK2, OMIM 602544), have been strongly implicated in the identification of damaged mitochondria for degradation via mitophagy [76]. PINK1 is a serine/threonine kinase localized to both mitochondria and the cytosol [77]. It accumulates on the outer membrane of damaged mitochondria, following a reduction in ∆Ѱm, and recruits Parkin to trigger the engulfment of mitochondria by autophagosomes [78]. The recruitment of Parkin requires PINK1-mediated phosphorylation of both Parkin and ubiquitin, which activates a ubiquitin phosphorylation cascade for mitophagy adapters. This feed-forward amplification loop leads to further recruitment and ubiquitylation-activation of Parkin so that the engulfment can proceed [79]. However, several studies have described a Parkin-independent mitophagy pathway associated with other E3 ubiquitin ligases including Glycoprotein 78 (Gp78) [80] and Ariadne RBR Ubiquitin E3 ligase 1 (ARIH1) [81].

### 3.2. Mitochondrial Biogenesis

The constant generation of new mitochondria is a key process for the maintenance of a healthy mitochondrial population with advancing age. The fact that the mitochondrial genome has limited protein coding capacity determines the dependence of mitochondrial biogenesis on synchronized transcription activity from both the mitochondrial and nuclear genomes. This provides building blocks which aid the replacement of mitochondria or can be inserted into remaining functional mitochondria [82]. Central to the regulation of mitochondrial biogenesis is the nuclear transcription cascade, mediated by peroxisome proliferator-activated receptor γ (PPARγ) coactivator 1α (PGC-1α) [83,84]. PGC-1α and its downstream cofactors nuclear respiratory factors (NRF1, 2) largely contribute to the regulation of transcription initiation of all nuclear-encoded RC proteins that are expressed by the nuclear genome. TFAM and dimethyladenosine transferase 2 (TFB2M) can then act in response to intracellular oxidative stress [85,86]. Reduction in the mRNA level of nuclear-encoded RC genes that are responsive to PGC-1α has been identified in individual DA neurons of PD patients, and in neurons in regions with subclinical PD-related Lewy body neuropathology [87]. The transcriptional decline in genes that control mitochondrial biogenesis and oxidative energy metabolism, including RC related genes and some cytosolic ribosomal genes (related to protein synthesis), are altered with advancing age [88,89]. This is supported by the discovery of downregulated AMP- activated protein kinase (AMPK) signalling, a key player in the control of PGC-1α expression, alongside decreased mitochondrial biogenesis in aged individuals [90]. These data strongly indicate the contribution of impaired biogenesis to the accumulation of mitochondrial dysfunction during ageing and the neurodegenerative process.

PGC-1α and its transcriptional control is critical for maintaining neuron function. Loss of dopaminergic neurons and striatal degeneration have been demonstrated in animal models where the PGC-1α gene is silenced or knockout [57,91,92]. Several ex vivo and in vivo studies have demonstrated the beneficial effects of PGC-1α overexpression and the corresponding increase in transcription activities in the rescue of dopaminergic neuronal loss induced by MPTP [93], rotenone, or alpha-synuclein mediated toxicity [94,95,96]. In addition to enhancing mitochondrial biogenesis, PGC-1α is also capable of activating the expression of antioxidant enzymes in response to oxidative stress [97,98] and protecting DA neurons against neuroinflammation [99]. However, there is still a lack of consensus on the value of upregulating PGC-1α expression as a PD therapeutic intervention. An increase in PGC-1α expression is not necessarily associated with an activation of its target cofactors that facilitate mitochondrial biogenesis [40]. Major alterations in energy metabolism that impair normal neuronal function have been found in a study of long term overexpression of PGC-1α in an alpha-synuclein mutant mouse, carrying the A53T mutation [33]. However, mitochondrial hyperactivity and increased ROS production were also described in this model, and may also contribute to the severe DA neuronal loss.

Shin et al. first identified a link between Parkin and PGC-1α in the regulation of PGC-1α expression via a Parkin interacting substrate, PARIS (also known as zinc finger protein 746, ZNF746). Parkin mediates the proteasomal degradation of PARIS. In the absence of Parkin, PARIS is bound to the promoter of PGC-1α gene and suppresses its expression [100]. This causes declines in mitochondrial respiratory capacity and mitochondrial mass, ultimately leading to dopaminergic neuronal death [33,34,101]. The parkin-mediated PARIS-dependent control of mitochondrial biogenesis, adds further evidence that impaired mitochondrial turnover contributes to the neuronal loss in PD.

It is hypothesized that mitochondrial turnover is slowed in aged and PD neurons due to defective nuclear transcriptional control of mitochondrial proteins. Alongside this, there is a decline in the clearance of dysfunctional mitochondria that occurs in coordination with a decrease in biogenic activity and, as a result, mutant mitochondria accumulate in the neurodegenerative process. Evidence for a decline in mitophagy during ageing has been highlighted in several recent reviews [102,103,104,105]. The abnormal accumulation of alpha-synuclein in PD neurons may put an additional burden on protein degradation pathways, increasing the vulnerability of SN neurons to mitochondrial dysfunction and pushing these neurons toward early cell death (reviewed in [106]).

### 3.3. Familial PD Associated Genes Related to Mitochondrial Turnover

#### 3.3.1. PINK1 and Parkin

Among many genes that are associated with familiar PD, *PINK1* and *Parkin* are highlighted due to their substantial involvement in mitochondrial maintenance. Although the PINK1/Parkin pathway is the key mediator of the process of mitophagy, recently more attention has been given to their regulatory roles in neuronal mitochondrial dynamics via their interactions with the Mitochondrial Rho GTPase1 (MIRO)/Milton complex (see below). In addition, the association of PINK1 and Parkin with the removal of mitochondrial proteins and impaired mitochondrial biogenesis has been suggested (reviewed in Reference [107]).

PINK1 has been suggested to also be important for the mitochondrial unfolded protein response (UPR^mt^). The UPR^mt^ was first reported in cell lines with a mutant form of ornithine transcarbamylase (OTC) [108], which led to accumulation of a misfolded form of the protein which subsequently drove expression of mitochondrial quality control genes. Believed to be a stress response to damaged mitochondrial proteins and hence dysfunction, alterations in the UPR^mt^ pathway may be of pathogenic importance in PD. For example, PINK1 has been proposed to be involved in this pathway through its interaction with HTRA serine peptidase 2 (HTRA2, PARK13, OMIM 606441) [109] and tumor necrosis factor receptor-associated protein 1 (TRAP1) [110]. Importantly, mutations in the genes encoding both these proteins have been associated with the development of PD [111,112]. In addition, the brains of those individuals carrying *PINK1* mutations show decreased levels of HTRA2 phosphorylation [109], while the brains of mice with a knockout of *HTRA2* show an accumulation of misfolded proteins within the mitochondria leading to reduced respiratory function and neurodegeneration [113]. TRAP1 is a mitochondrial chaperone protein, whose knockout in *Drosophila* causes an age-related decrease in climbing ability and dopamine levels in the brain, increased sensitivity to mitochondrial toxins including rotenone and a decrease in ATP levels [114]. It is also a PINK1 effector whose expression in PINK1 deficient *Drosophila* rescues morphological and mitochondrial defects and is capable of reducing dopamine deficits [114,115]. Given the importance of the UPR^mt^ for the maintenance of mitochondrial function, this pathway may prove to be an important therapeutic target. Upregulation of the UPR^mt^ for example, may allow mitochondrial function to be improved through either degradation of unfolded mitochondrial proteins or increases in the upstream signalling cascade.

#### 3.3.2. DJ-1 and LRRK2

The functions of DJ-1 (PARK7, OMIM 606324) and leucine-rich repeat kinase 2 (LRRK2, PARK8, OMIM 609007) are less well characterized in terms of mitochondrial health. Downregulation of DJ-1 causes multiple mitochondrial abnormalities, including increased mitochondrial fragmentation and reduced ∆Ѱm and connectivity [116,117]. Interestingly, this effect can be blocked by overexpression of Parkin and PINK1 [118]. A recent study using induced pluripotent stem cells (iPSC)-derived neurons reports the beneficial effect of LRRK2 in the removal of MIRO, and thus enhanced arrest of damaged mitochondria. Pathogenic *LRRK2* mutations disrupt this function, speeding up mitochondrial mobility and consequentially slowing the initiation of mitophagy [119].

#### 3.3.3. ATP13A2 and GBA

Novel mutations in *ATP13A2* (ATPase type 13A2, PARK9, OMIM 610513) have been identified as a genetic cause of a rare juvenile-onset form of PD, named Kufor–Rakeb syndrome [120,121]. *ATP13A2* encodes an isoform of type P5B ATPase, which functions as a lysosomal ATPase transporter. It is believed to play critical roles in the regulation of vesicular transportation [122], endolysosomal activity [123,124], and glycolysis [125]. Deficiency of ATP13A2 causes lysosomal defects, and thus affects cation homeostasis, for example Zn^2+,^ leading to impaired mitochondrial respiratory function and defective mitophagy [89,122]. A recent study also found gliosis in multiple brain regions of heterozygous *ATP13A2* knockout mice, suggestive of the neuroinflammation which occurs in the early stages of PD development [123]. Another heterozygous mutation in the gene that encodes the lysosomal enzyme glucocerebrosidase (*GBA*, OMIM 606463) has also been identified as a powerful genetic risk factor for PD [126,127]. Those PD patients who carried a *GBA* mutation tended to have an earlier age of onset and an increased risk of developing dementia compared to idiopathic PD patients [128]. Together, this evidence highlights that lysosomal alterations may be crucial to the pathogenesis PD, exacerbating the accumulation of mitochondrial damage and abnormal protein aggregation [129].

### 3.4. Does Mitochondrial Turnover Drive the Development of Neurodegeneration in PD?

Although the process is still not clearly defined in neurons, data suggests that the degradation of dysfunctional mitochondria occurs through the autophagy related pathway, mitophagy. A number of studies have suggested that there is a decline in mitophagy with age (reviewed in References [102,103]). Furthermore, many of the genes that control the degradation of mitochondria through this pathway have been linked to ageing and lifespan in model organisms (reviewed in [130]). A decline in lysosome function has also been shown to occur with advancing age, mainly through the accumulation of lipofuscin within these organelles [131]. However, the fact that many of the genes responsible for familial PD have been shown to have important roles in autophagy or lysosomal pathways suggests that this is an important driver of the pathogenesis of PD. Alterations in the efficiency of such pathways will clearly exacerbate mitochondrial dysfunction, allowing defective mitochondria to accumulate. Mitochondrial dysfunction itself may also increase oxidative damage to proteins and organelles which may overwhelm these systems in PD, leading to neuronal loss. Again, we are faced with a vicious circle of damage, whereby mitochondrial dysfunction may be caused by defects in genes such as *ATP13A2*. However, lysosomal dysfunction may increase oxidative stress, which would then cause further mitochondrial and cellular damage.

## 4. Mitochondrial Dynamics, Transport, and Distribution

The precise distribution of mitochondria within neurons is a fundamental aspect of the correct function of these cells. Neuronal communication through electrical impulse and chemical synapses is fundamental for brain function, and, in PD, a loss of synapses has been shown to precede the loss of cell bodies from the substantia nigra. This decline in synaptic terminal density may begin decades before the first symptoms appear in affected individuals (reviewed in Reference [132]). Therefore, understanding the causes of this synaptic loss may be key to unlocking new therapies for PD.

As described above, mitochondrial production relies on signalling between the nucleus and the mitochondria, while the degradation of dysfunctional mitochondria requires the interaction of a number of proteins and signalling pathways that we are only just beginning to understand. Mitochondrial biogenesis generally occurs at the soma, in close proximity to the nucleus, although evidence has suggested that mtDNA replication may occur within axons at sites distant from the cell body [133,134,135]. The degradation of damaged mitochondria has generally been thought to occur within the cell body, since this was thought to be the only location of lysosomes, though recently this convention has also been challenged [136]. Data has also suggested that mitochondria may be donated to and removed from neurons by astrocytes, with such processes occurring in response to cellular stress [137,138]. Therefore, neurons require processes which allow mitochondrial movements to be responsive to changes within the local and intracellular environments.

Mitochondria perform a variety of roles within neurons to support their function. They provide localized ATP to regions of the neuron which are particularly energy demanding (including the nodes of Ranvier and pre-synaptic terminals) and buffer cytosolic calcium. These two functions of mitochondria support the release and recycling of synaptic vesicles and the maintenance of electrochemical gradients, which are intimately dependent on each other since they both rely on calcium signalling. Therefore, as the main organelles which support these processes, there is a need for mitochondria to be dynamic and responsive in their distribution. The movement and localization of mitochondria can be affected by their function. A loss of mitochondrial membrane potential, for example, has been proposed to drive directional mitochondrial movement. Vice versa, incorrect positioning of mitochondria within neurons may have a detrimental effect on the health and function of neurons (reviewed in [139,140]).

### 4.1. Fission and Fusion

The processes of fission and fusion control the network connectivity of mitochondria within neurons. These processes allow the creation or destruction of a mitochondrial syncytium, in which mitochondrial contents may be shared, or dysfunctional mitochondria partitioned for destruction. The degradation of dysfunctional mitochondria through mitophagy and the manipulation of this process has been suggested to be an important therapeutic avenue for many degenerative diseases [141]. While many of the specifics of exactly how mitochondria are detected as being targets for degradation remain unclear, it is likely that the fission of such organelles from the network will be a key process [142]. Fragmentation of the mitochondrial network occurs rapidly following the loss of mitochondrial membrane potential, and if this loss cannot be restored, fragmented mitochondria lose their “fusion” capabilities and are degraded [142]. Many of the proteins that control these two process have been characterized and alterations in these processes have been detected in a number of models of familial PD, yielding differences in the size of mitochondria and their ultrastructure [143]. Moreover, a loss of dynamin-1-like protein (DRP1), a key protein in the fission machinery within dopaminergic neurons, causes a Parkinson’s like phenotype in affected mice due to degeneration of SN neurons. A staggering 85% of SN neurons had been lost by the age of 1 month in these animals and this loss was due to depletion of the axonal mitochondrial population [144]. Furthermore, mutations in *OPA1* (OMIM 605290) have been linked with parkinsonism, including alterations upon DAT-SPECT scanning [145]. The two heterozygous mutations reported in this paper also caused alterations to the structure and function of mitochondria [145]. In SHSY5Y cells, the overexpression of OPA1 has been shown to protect against the ultrastructural abnormalities induced by treatment with MPP^+^, although interestingly this was not related to a reduction of fission [146]. The fusion of mitochondria has been further linked with PD by the finding that mitofusin 1 (MFN1) and MFN2 are ubiquitinated in a Parkin/PINK1 dependent manner [147,148]. The ubiquitination of mitochondrial proteins is a key step in their identification for degradation through mitophagy. Further details of this process have emerged showing that PINK1 phosphorylates MFN2, and this phosphorylation then recruits Parkin, which then ubiquitinates the protein [149]. This study also showed that in cardiomyocytes, a deficiency of MFN2 impedes mitophagy, leading to the accumulation of abnormal mitochondria and respiratory dysfunction [149]. The ubiquitination of the mitofusin proteins is impeded in fibroblasts from individuals with pathogenic *PINK1* or *Parkin* mutations [150]. Interestingly, other proteins important for the fission and fusion of mitochondria are not affected by mutations within these genes [150]. The ubiquitination of the mitofusions will act not only to identify mitochondria for degradation, but will also ensure that such mitochondria are unable to rejoin the mitochondrial network. In addition, as described above, many of the neurotoxins used to generate experimental models of Parkinson’s Disease, including rotenone and MPTP, cause the fragmentation of the mitochondrial network, most likely due to their inhibitory effects on mitochondrial complex I [151,152,153]. In fact, their toxicity might be directly related to their effect on the balance of mitochondrial biogenesis and fission/fusion [154].

### 4.2. Mitochondrial Distribution

The correct positioning of mitochondria within neurons is required for localized supply of ATP and calcium buffering. By performing these essential tasks mitochondria become integral to the functioning and survival of neurons. The provision of ATP is crucial for the support of neuronal electrical activity, and in line with this, mitochondria are found clustered at sites with high energy demands, for example, at the Nodes of Ranvier in myelinated axons. Their location at such sites supports the function of ion channels such as the Na^+^/K^+^ ATPase, which is responsible for the restoration of resting membrane potential following an action potential. Furthermore, their distribution changes to mirror alterations in channel distribution, for example, following demyelination [155]. In addition, the positional holding of mitochondria at these locations changes in response to electrical activity. A decline or arrest in electrical activity will increase mitochondrial mobility away from nodes, while conversely, depolarization leads to a movement of mitochondria into these areas to provide ATP to repolarize the membrane (reviewed in Reference [156]).

Localized reliable sources of ATP are also essential for synaptic function. Within the presynaptic terminal, mitochondrial ATP is required for an array of processes, including vesicle recycling, exo and endocytosis of vesicles, and the loading of recycled vesicles with neurotransmitter (reviewed in Reference [157]). As crucial as this energy provision is the ability of mitochondria to buffer calcium (see above). The release of neurotransmitter from presynaptic terminals relies on a number of processes which are modulated by calcium signalling. As such, the concentration of calcium within the presynaptic terminal must be tightly regulated, to control the quantal release of neurotransmitter, the priming of terminals for the next depolarization, and changes in synaptic strength. Calcium buffering might be particularly important for the neurons of the substantia nigra, given their pace-making activity. Blockage of the calcium channel, which governs this activity, forces the neurons to utilize sodium channels to maintain this low frequency activity, which also protects neurons against mitochondrial dysfunction caused by rotenone treatment [158].

Mitochondria can be recruited to synapses, though not all synapses contain mitochondria. Synapses devoid of mitochondria have been found in a number of cell types, including hippocampal neurons, primary cortical neurons, and the DA neurons of the nigrostriatal pathway [159,160,161]. The number of these “empty” synapses was recently shown to decline in the presence of dopaminergic neuronal degeneration in PD [162]. This suggests two interesting hypotheses, either synapses which lack mitochondria show an increased vulnerability in PD and are the first to be lost, or that in the presence of degenerating neighbors, neurons can populate such synapses with mitochondria and the replenishment with ATP activates them as part of an adaptation to maintain transmission to those post-synaptic sites. In neurons with complex architecture, such as the neurons of the nigrostriatal pathway which have been suggested to have a huge number of pre-synaptic terminals, it may seem obvious that they would not utilize the full synaptic complement but keep some “in reserve” in case of loss of a proportion.

### 4.3. Mitochondrial Transport

The correct distribution of mitochondria and the maintenance of a healthy functional population within DA neurons relies on efficient transport from sites of genesis or to sites of degradation. The movement of mitochondria along axons relies on motor proteins and microtubules with different proteins mediating retrograde (towards the cell body) and anterograde (towards the terminus) transport. The directionality of mitochondrial movements is controlled by different sets of motors with dynein motors controlling retrograde transport, while anterograde movements are driven by kinesin motors (reviewed in Reference [157]). The transport of mitochondria over long distances within neurons is much more complicated than this and additional protein interactions are required namely between trafficking kinesin binding proteins (TRAKs), kinesin heavy chain isoform 5 (KIF5) and dynein, and the mitochondrial proteins MIRO1 and 2. These outer mitochondrial membrane proteins are responsible for the interaction of mitochondria with these molecular motors. An in-depth discussion of the mechanisms of mitochondrial transport is out of the realm of this review, however, there are a number of excellent reviews which cover this in detail [139,156,157,163].

Previous work has suggested that mitochondria are transported towards the cell body when they have lost their membrane potential, a key signal for their sequestration in autophagosomes, although other studies have been unable to replicate this [164,165]. Many of the interactions involved in these processes have been well characterized, as have the patterns of movement exhibited by mitochondria under normal and disease conditions. In line with the fact that mitochondria are predominantly held at sites where they are required to provide localized ATP or calcium buffering, the majority of mitochondria within neurons are stationary, with only 10–20% of mitochondria being motile in hippocampal neurons [160,166], with over 90% reported as being stationary in cortical and spinal neurons [167,168,169]. Those mitochondria move through showing a variety of velocities and movements, with some moving in one direction with others seemingly wobbling back and forth. These mitochondrial movements also change with maturation of neurons, with more mitochondria becoming immobile and enriched at sites such as synapses [168].

The docking of mitochondria at discrete sites of the neuron relies on calcium. Localized elevated calcium levels cause mitochondrial stalling. In cultured neurons, mitochondrial mobility is decreased by increasing calcium influx [159,170]. The ability of calcium to cause mitochondrial immobility relies on MIRO and the binding of calcium to the proteins two EF hands. Mutations within these domains leading to impaired calcium binding have been shown to prevent the ability of mitochondria to dock (reviewed in Reference [171]). The importance of calcium for the transport and mobilization of mitochondria is particularly interesting given the importance of calcium for the pace-making activity exhibited by dopaminergic SN neurons (reviewed in Reference [56]). Many studies have investigated mitochondrial trafficking in mouse and cell culture-based models of PD [172,173]. For example, the complex I inhibitor MPP^+^ alters mitochondrial directionality, increasing the speed of retrograde movement and decreasing the opposing movements. Furthermore, this study also showed that of the mitochondria that were moving, MPP^+^ caused the stalling of over 50% of these. Interestingly, this study also uncovered that mitochondria within DA neurons were much smaller than those within non-DA axons. This reduction in size in DA neurons may suggest that there are inherent differences in the levels of fission and fusion between DA neurons and other neuronal populations. Differences in transport in these cells may also impede the delivery of mitochondria to synapses and other discrete sites within the neurons [172]. Furthermore, in the DA neurons of the MitoPark mouse (conditional TFAM knockout) the supply of mitochondria to axonal terminals is impaired due to reduced anterograde transport [173].

### 4.4. Familial PD Genes and Their Association with Mitochondrial Dynamics

The importance of many of the genes associated with PD for mitochondrial dynamics lies in the ability of the proteins they encode to affect the transport of mitochondria. Of all the proteins which have been associated with familial PD, the ones most associated with mitochondrial dynamics, obviously given their roles in mitophagy, are PINK1 and Parkin. Initially thought of as key mediators of mitochondrial degradation through mitophagy, the impact and responsibility of Parkin and PINK1 on the maintenance of mitochondrial integrity is now believed to be more complex. Recent studies have also proposed roles for these two proteins in the mitochondrial unfolded protein response, in the modulation of mitochondrial fission and fusion, and finally in mitochondrial transport and trafficking (reviewed in depth in Reference [174]).

Early studies showed that PINK1, a mitochondrial outer membrane protein, was able to recruit Parkin to mitochondria upon mitochondrial depolarization with the uncoupler carbonyl cyanide m-chlorophenyl hydrazine (CCCP) [78,175]. We now know that PINK1 actively recruits Parkin to mitochondria and phosphorylates it, Parkin is then able to ubiquitinate outer mitochondrial membrane proteins signalling the mitochondrion for destruction. The mitochondrion is then enveloped by an autophagosome and trafficked to the lysosome (reviewed in Reference [176]). *PINK1* and *Parkin* mutations in animal models have been shown to lead to both mitochondrial structural and functional changes, which are often causative of the DA neuron degeneration in these models [177,178]. Parkin overexpression has the ability to rescue many of the phenotypic changes detected in *PINK1* mutants, including the mitochondrial defects and DA loss [177]. Furthermore, the expression of Parkin in cell lines has been shown to drive expression of a number of mitochondria associated proteins, including a number important for mitochondrial dynamics, trafficking, and the ubiquitin proteasome [179].

PINK1 and Parkin have also been linked to mitochondrial dynamics and trafficking through their interaction with a number of proteins essential to these processes. For example, overexpression of PINK1 or Parkin in neurons has been shown to decrease bidirectional mitochondrial movements. PINK1 phosphorylates MIRO, which then causes Parkin mediated degradation and kinesin release [180,181]. The knockdown of PINK1 has been shown to promote anterograde mitochondrial transport in *Drosophila* motor neurons, while conversely, a loss of MIRO in Hela cells was linked to perinuclear mitochondrial clustering and mitochondrial degradation by mitophagy [180]. PINK1 expression in mitochondria has been found to be linked to expression of both MIRO and Milton, with which it forms a complex. Furthermore, the interaction with these proteins can drive the association of PINK1 with mitochondria even in the absence of a PINK1 mitochondrial targeting sequence [182]. Finally, Parkin expression has been found to decrease the amount of mitochondrial movement in hippocampal neurons. This change in mitochondrial movement was also found to be associated with a reduction in axonal mitochondrial density [181].

### 4.5. Do Mitochondrial Dynamics Drive the Development of Neurodegeneration in PD?

Together these data suggest that many of the proteins that are known to be integral to the development of PD have now been shown to have crucial roles in maintaining the dynamics, distribution, and transport of mitochondria within neurons. We are now beginning to understand more about these dynamic aspects of mitochondria due to improved imaging techniques and models. It is clear, however, that the correct distribution of mitochondria within neurons is as important to their function as the interconnectivity of these organelles. Furthermore, it is not only the movements of functional mitochondria which need to be monitored and controlled, but also the localisation and transport of those which are dysfunctional and require degradation. Disruption of these processes in many models has been linked with impaired neuronal function or even neurodegeneration. With advancing age and in PD the neurons of the SN do accumulate mitochondria which are dysfunctional and may therefore be unable to provide the required level of ATP. Failure to remove these mitochondria from important sites such as the synapse will then lead to further detrimental changes (as described above) and neuronal loss.

## 5. Mitochondria and Protein Aggregation

Protein aggregation in Parkinson’s disease is predominantly driven by alpha-synuclein, a small protein with a propensity to aggregate into oligomeric and fibrillar forms upon damage or mutation. This small protein has been proposed to function to support synaptic transmission, with proposed roles in vesicular packaging, synaptic vesicle trafficking and synaptic plasticity (reviewed in Reference [183]). The interaction of alpha-synuclein with mitochondria has been studied in depth over recent years, particularly since it has been suggested that it may be imported into mitochondria and cause inhibition of complex I [184]. The specific interaction of alpha-synuclein with mitochondrial complex I causes a reduction in its activity. This inhibition can be driven by both wild-type and mutant alpha-synuclein, and by oligomeric and fibrillar forms of the protein [45,185,186]. This functional disruption of mitochondria has most recently been shown to be also driven by an interaction of alpha-synuclein with complex V (ATP synthase) causing alterations in mitochondrial morphology, accompanied by an opening of the permeability transition pore, an increase in oxidative stress, and ultimately, neuronal death [45]. Recent work has highlighted that this capacity to interact with mitochondria may also impact on the ability of mitochondria to import essential proteins from the cytosol [184,187,188]. Alpha-synuclein has been shown to interact with the TOM (translocase of the outer membrane) complex in both tissue and culture-based systems. A blockage of import in this way has previously been shown to be sufficient to drive nigrostriatal degeneration [189].

Alpha-synuclein has been shown to cause fragmentation of the mitochondrial network in a number of models [190,191,192]. This fragmentation is DRP1 independent, occurs in the presence of both wild-type and mutant forms of the protein, and has been shown to precede the loss of mitochondrial function and neuron death. Interestingly, it has been suggested that the protein has no effect on the structure of the endoplasmic reticulum, the Golgi, or their interactions with the mitochondria. However, a recent study has shown that alpha-synuclein does interact with VAPB, an integral ER protein. This interaction then weakens the association of mitochondria with the ER, causing alteration in calcium buffering and the production of ATP [193]. The effect of alpha-synuclein on the structure of the mitochondria extends beyond merely affecting their fission and fusion with alterations to the ultrastructure of mitochondria also reported. Kamp et al. showed that PINK1, Parkin, and DJ-1 over expression was sufficient to be able to rescue the effect of alpha-synuclein on mitochondrial dynamics [191].

Alpha-synuclein has been suggested to be important for synaptic vesicle trafficking for many years, but recently, mounting evidence has suggested that it also affects the movement of mitochondria around neurons. In neurons exposed to alpha-synuclein, the balance between retrograde and anterograde transport is shifted. Wild type neurons showed a skewed preference for anterograde movement to maintain the axonal mitochondrial populations, and this preference was lost when alpha-synuclein expressed [194]. Kymograph analysis in a zebrafish model confirmed that alpha-synuclein reduced anterograde mitochondrial movements along axons and that the mitochondria within these axons, spent more time in a paused/stationary state [96]. The ability of alpha-synuclein to interrupt mitochondrial trafficking in this manner has been studied in depth, and is driven by its interaction with microtubules and cytoskeletal elements. Alpha-synuclein is capable of binding to a number of these proteins, including kinesin family member 5 (KIF5A), microtubule-associated protein 2 (MAP2), and Tau, with wild type synuclein showing the strongest binding to these proteins, with oligomers and fibrils showing weaker interactions [195]. The effect of alpha-synuclein on microtubule stability affects the distribution of mitochondria to neurites which would have detrimental effects on neuronal health and survival.

### Does the Interaction of Alpha-Synuclein with Mitochondria Drive the Development of Neurodegeneration in PD?

Alpha-synuclein, of all the proposed contributors to Parkinson’s pathogenesis, perhaps affects the most aspects of mitochondrial function. Alpha-synuclein has been shown to impact mitochondrial function, dynamics, transport, and protein import. Considering that alpha-synuclein aggregation is still one of the pathological hallmarks of PD, this would certainly suggest that in the presence of mitochondrial defects, it may lead to neurodegeneration. However, it is important to consider that studies have reported that Lewy bodies accumulate in neurons which do not have mitochondrial deficiencies, and that not all alpha-synuclein models recapitulate all of the characteristics of PD. Mitochondrial dysfunction, caused by alpha-synuclein or mtDNA mutations, may cause oxidative stress which will further damage alpha-synuclein, causing it to misfold into oligomeric forms. These alpha-synuclein aggregates are then capable of exacerbating mitochondrial dysfunction, as well as interfering with mitochondrial turnover and transport. This would lead to incorrect distribution of mitochondria, impaired calcium buffering, and ATP production increasing the sensitivity of these neurons to loss in Parkinson’s.

## 6. Conclusions

Mitochondrial dysfunction, initially detected as Complex I deficiency, is found within the neurons of the SN, the population of neurons whose loss leads to the development of the motor symptoms associated with this disease. This deficiency may be caused by mtDNA deletions, by alpha-synuclein oligomers, or environmental toxins. A loss of mitochondrial function impedes ATP production and calcium buffering, key processes in the function of these neurons, and exacerbates oxidative stress. Such mitochondria should be targeted for degradation through the mitophagy pathway and replenished with new functional organelles through the PGC1α biogenesis pathway, both of which are affected by advancing age and have been shown to be linked to Parkinson’s disease. Furthermore, dysfunctional mitochondria require transport to lysosomes and away from energy demanding sites. Recent evidence has also hinted that the dynamic processes that control the distribution and structure of mitochondria are also linked with PD. All these processes have the ability to cause further accumulation of defective mitochondria if they are themselves impaired.

**Figure 2:**
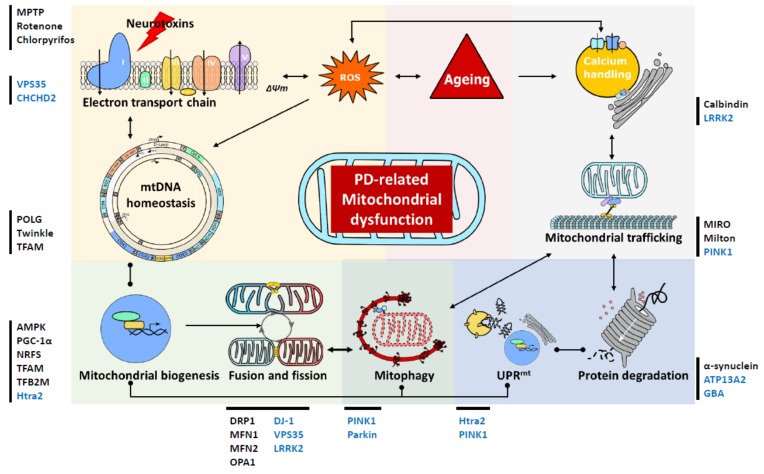
Schematic presentation of mitochondrial involvement in the pathogenesis of Parkinson’s disease (PD). This diagram serves to highlight the complex links between the changes in mitochondrial homeostasis, turnover, quality control and trafficking in cases of PD. These mitochondrial alternations are also intricately associated with the ageing process and impairments of the ubiquitin protease system that are attributed to Lewy body pathology. Lines with dots represent interactive effects, lines with arrows represent regulatory effects. Genes associated with familial PD are shown in blue, while we have also highlighted other proteins and toxins, recently been associated with PD, which impact on mitochondrial function. MPTP: 1-methyl-4-phenyl-1,2,3,6-tetrahydropyridine; VPS35: vacuolar protein sorting 35; CHCHD2: coiled-coil-helix-coiled-coil-helix domain containing 2; TFAM: mitochondrial transcription factor A; AMPK: AMP-activated protein kinase; PGC-1α: peroxisome proliferator-activated receptor γ (PPARγ) coactivator 1α; NRF: nuclear respiratory factors; TFB2M: dimethyladenosine transferase 2; DRP1: dynamin-1-like protein; MFN: mitofusin; LRRK2: Leucine-rich repeat kinase 2; PINK1: phosphatase and tensin homolog (PTEN)-induced putative kinase 1; GBA: lysosomal enzyme glucocerebrosidase; MIRO: mitochondrial Rho GTPase1; ∆Ѱm: mitochondrial membrane potential; ROS: reactive oxygen species; mtDNA: mitochondrial DNA; UPR^mt^: the mitochondrial unfolded protein response.

Therefore, it is clear that alterations in mitochondrial function are important for neuronal survival and are a key driver of neuronal loss in Parkinson’s disease (Figure 2). Since the initial description of Complex I deficiency in this disorder, our understanding not only of the complexities of mitochondria but also of the pathways and proteins whose function has an impact on, or is impacted by mitochondrial function, has dramatically increased. However, despite overwhelming evidence to suggest that the function of mitochondria is important for PD pathogenesis, the ability to identify the initial event in the cascade of changes that leads to neurodegeneration remains elusive. While here we have tried to categorize a number of proteins by their primary role (for example PINK1/Parkin as mitophagy proteins), it is becoming clear that many of these proteins have a role to play in a host of other pathways, while many of these also have differential consequences for the mitochondria (for example, the contribution of PINK1/Parkin to mitochondrial dynamics). While current techniques and models are driving this search forward, it is clear that the complex nature of the mitochondria and the role they play in a host of cellular pathways means that they have a large impact on a number of processes (energy provision, calcium buffering, ROS production, etc.). However, since their function is also impacted by changes in many of these pathways, it would seem that for every link between mitochondrial function and a pathway (for example, mitochondrial dynamics), a contrary relationship will then be found between the two processes. Understanding more about these interactions will uncover novel drugs which can modulate them not only to preserve mitochondrial function, but also to protect these organelles against damage and damaging moieties, driving and supporting the hunt for neuroprotective treatments.
